# Supplementary material for: Coordination of tissue cell polarity by auxin transport and signaling
Source: eLife. 2019 Dec 3;8:e51061. doi: 10.7554/eLife.51061 (PMC6890459; doi:10.7554/eLife.51061)
Supplement: Supplementary file 1. [file elife-51061-supp1.docx]

| **Key Resources Table** | | | | |
| --- | --- | --- | --- | --- |
| **Reagent type (species) or resource** | **Designation** | **Source or reference** | **Identifiers** | **Additional information** |
| Strain (*Arabidopsis thaliana*) | PIN1::PIN1:YFP | PMID: 16424342 |  |  |
| Strain (*Arabidopsis thaliana*) | *gn-13* | ABRC; this paper; PMID: 12893945 | SALK_045424 | Contains a T-DNA insertion after +2835 of *GN* (AT1G13980) |
| Strain (*Arabidopsis thaliana*) | PIN1::PIN1:GFP | PMID: 14651850 |  |  |
| Strain (*Arabidopsis thaliana*) | *gn-18* | ABRC; this paper; PMID: 12893945 | SALK_026031 | Contains a T-DNA insertion after -1047 of *GN* (AT1G13980) |
| Strain (*Arabidopsis thaliana*) | *gn^fwr^* | PMID: 23390202 | *fwr* |  |
| Strain (*Arabidopsis thaliana*) | *gn^B/E^* | PMID: 14681187 |  |  |
| Strain (*Arabidopsis thaliana*) | *gn^R5^* | PMID: 14681187 |  |  |
| Strain (*Arabidopsis thaliana*) | *gn^van7^* | PMID: 10887076 | *van7*; *emb30-7*; |  |
| Strain (*Arabidopsis thaliana*) | *gn^van7+fwr^* | This paper |  | *gn^van7^* (-2127 to +5388; primers: “GN Fwd NotI” and “GN Rev NotI”) containing the *fwr* mutation (primers: “fwr-mutagenesis F” and “fwr-mutagenesis R”) |
| Strain (*Arabidopsis thaliana*) | *gn^SALK_103014^* | PMID: 23390202 |  |  |
| Strain (*Arabidopsis thaliana*) | *gn^emb30-8^* | ABRC; PMID: 2423279, 24388525 | *emb30-8* |  |
| Strain (*Arabidopsis thaliana*) | PIN2::PIN2:GFP | PMID: 15659621 |  |  |
| Strain (*Arabidopsis thaliana*) | PIN3::PIN3:GFP | PMID: 20110326 |  |  |
| Strain (*Arabidopsis thaliana*) | PIN4::PIN4:GFP | PMID: 2711952, 29192026 |  |  |
| Strain (*Arabidopsis thaliana*) | PIN7::PIN7:GFP | PMID: 29192026 |  |  |
| Strain (*Arabidopsis thaliana*) | *pin1-1* | ABRC; PMID: 9856939, 23437008 |  | WT at the *TTG1* (AT5G24520) locus |
| Strain (*Arabidopsis thaliana*) | *pin1-134* | PMID: 9856939; this paper |  | Derived from *Atpin1::En134*; contains a 4-bp (AATT) insertion between +134 and +135 of *PIN1* (AT1G73590), resulting in a stop codon after amino acid 62. |
| Strain (*Arabidopsis thaliana*) | *pin3-3* | PMID: 11845211 |  |  |
| Strain (*Arabidopsis thaliana*) | *pin4-2* | PMID: 11893337 |  |  |
| Strain (*Arabidopsis thaliana*) | *pin7^en^* | PMID: 14651850, 15635403, 14614497; this paper |  | Contains a transposon insertion after +707 of *PIN7* (AT1G23080) |
| Strain (*Arabidopsis thaliana*) | *pin2* | ABRC; PMID: 7768447, 9679062 | *eir1-1* |  |
| Strain (*Arabidopsis thaliana*) | *toz-1* | PMID: 17616738 |  |  |
| Strain (*Arabidopsis thaliana*) | *mp^G12^* | PMID: 9482737, 24281793 |  |  |
| Strain (*Arabidopsis thaliana*) | *pin6* | ABRC; PMID: 23437008 |  |  |
| Strain (*Arabidopsis thaliana*) | *pin8-1* | ABRC; PMID: 22540348 |  |  |
| Strain (*Arabidopsis thaliana*) | ABCB1::ABCB1:GFP | PMID: 1833751, 18787070 |  |  |
| Strain (*Arabidopsis thaliana*) | ABCB19::ABCB19:GFP | PMID: 1833751, 18787070 |  |  |
| Strain (*Arabidopsis thaliana*) | *abcb1* | ABRC; PMID: 15908594 | *pgp1-100* |  |
| Strain (*Arabidopsis thaliana*) | *abcb19* | ABRC; PMID: 15908594 | *mdr1-101* |  |
| Strain (*Arabidopsis thaliana*) | *twd1* | ABRC; PMID: 14730066 | *ucu2-4* |  |
| Strain (*Arabidopsis thaliana*) | *aux1-21*;*lax1*;*2-1*;*3* | PMID: 1862238, 1834709, 1548610, 25617434 |  |  |
| Strain (*Arabidopsis thaliana*) | *aux1-355* | ABRC; this paper; PMID: 12893945 | SALK_020355 | Contains a T-DNA insertion after +631 of *AUX1* (AT2G38120) |
| Strain (*Arabidopsis thaliana*) | *lax1-064* | ABRC; this paper; PMID: 12893945 | SALK_071064 | Contains a T-DNA insertion after +814 of *LAX1* (AT5G01240) |
| Strain (*Arabidopsis thaliana*) | *axr1-3* | ABRC; PMID: 1983791, 8321287 |  |  |
| Strain (*Arabidopsis thaliana*) | *axr1-12* | ABRC; PMID: 1983791, 8321287 |  |  |
| Strain (*Arabidopsis thaliana*) | *axl* | ABRC; this paper; PMID: 1246872, 17655650 | SAIL_673_C11 | Contains a T-DNA insertion after +1390 of *AXL* (AT2G32410) |
| Strain (*Arabidopsis thaliana*) | *tir1-1*;*afb2-3* | PMID: 1881830, 2001875, 2156454, 2761844, 28760746 |  |  |
| Strain (*Arabidopsis thaliana*) | DR5rev::nYFP | PMID: 1627186, 23437008 |  |  |
| Sequence-based reagent | SALK_045424 gn LP | Integrated DNA Technologies, Inc. |  | TGATCCAAATCACTGGGTTTC |
| Sequence-based reagent | SALK_045424 gn RP | Integrated DNA Technologies, Inc. |  | AGCTGAAGATAGGGAATTCGC |
| Sequence-based reagent | LBb1.3 | Integrated DNA Technologies, Inc. |  | ATTTTGCCGATTTCGGAAC |
| Sequence-based reagent | Salk026031 LP gnp close | Integrated DNA Technologies, Inc. |  | TGAAAGAGACATGTCCTTCGG |
| Sequence-based reagent | Salk026031 RP gnp close | Integrated DNA Technologies, Inc. |  | GACACGTCTCGCTAAATCTCG |
| Sequence-based reagent | FWR for | Integrated DNA Technologies, Inc. |  | AAGAGCCAAGATCACAGCCTACTG |
| Sequence-based reagent | FWR REV2 | Integrated DNA Technologies, Inc. |  | GAGAGCACGCGCAAGCTGCAACAAG |
| Sequence-based reagent | van7 Hpa1 FP | Integrated DNA Technologies, Inc. |  | ATCCGTGCCCTTGATCTAATGGGAG |
| Sequence-based reagent | van7 Hpa1 RP | Integrated DNA Technologies, Inc. |  | CACTTTTCTTAGTCCTTGAACAAGCGTTAA |
| Sequence-based reagent | GN Fwd NotI | Integrated DNA Technologies, Inc. |  | TCTGCGGCCGCTCTAGAGGTGTGTATGATAATG |
| Sequence-based reagent | GN Rev NotI | Integrated DNA Technologies, Inc. |  | TTTGCGGCCGCTCTAGAAATCGAAATCCGTCTC |
| Sequence-based reagent | fwr-mutagenesis F | Integrated DNA Technologies, Inc. |  | GCTTGCGCGTGCTCTCATTTGGGC |
| Sequence-based reagent | fwr-mutagenesis R | Integrated DNA Technologies, Inc. |  | TGCAACAAAAATTCAGCTTGTAGAAACTTGCTTTCG |
| Sequence-based reagent | pin1-1 F | Integrated DNA Technologies, Inc. |  | ATGATTACGGCGGCGGACTTCTA |
| Sequence-based reagent | pin1-1 R | Integrated DNA Technologies, Inc. |  | TTCCGACCACCACCAGAAGCC |
| Sequence-based reagent | pin1-134 R mse-I | Integrated DNA Technologies, Inc. |  | CTCAGCTTCAGTTTCCAAAGGTTG |
| Sequence-based reagent | pin3-3 F | Integrated DNA Technologies, Inc. |  | GGAGCTCAAACGGGTCACCCG |
| Sequence-based reagent | pin3-3 R | Integrated DNA Technologies, Inc. |  | GCTGGATGAGCTACAGCTATATTC |
| Sequence-based reagent | PIN4 forw geno II | Integrated DNA Technologies, Inc. |  | GTCCGACTCCACGGCCTTC |
| Sequence-based reagent | PIN4en rev Ikram | Integrated DNA Technologies, Inc. |  | ATCTTCTTCTTCACCTTCCACTCT |
| Sequence-based reagent | en primer | Integrated DNA Technologies, Inc. |  | GAGCGTCGGTCCCCACACTTCTATAC |
| Sequence-based reagent | PIN7en forw Ikram | Integrated DNA Technologies, Inc. |  | CCTAACGGTTTCCACACTCA |
| Sequence-based reagent | PIN7en rev | Integrated DNA Technologies, Inc. |  | TAGCTCTTTAGGGTTTAGCTC |
| Sequence-based reagent | PIN7en rev Ikram II | Integrated DNA Technologies, Inc. |  | GGTTTAGCTCTGCTGTGGAGTT |
| Sequence-based reagent | eir1-1 F | Integrated DNA Technologies, Inc. |  | TTGTTGATCATTTTACCTGGGACA |
| Sequence-based reagent | eir1-1 R | Integrated DNA Technologies, Inc. |  | GGTTGCAATGCCATAAATAGAC |
| Sequence-based reagent | PIN6 spm F | Integrated DNA Technologies, Inc. |  | CATAACGAAGCTAACTAAGGGGTAATCTC |
| Sequence-based reagent | PIN6 spm R | Integrated DNA Technologies, Inc. |  | GGAGTTCAAAGAGGAATAGTAGCAGAG |
| Sequence-based reagent | Spm32 | Integrated DNA Technologies, Inc. |  | TACGAATAAGAGCGTCCATTTTAGAGTG |
| Sequence-based reagent | SALK_107965 LP | Integrated DNA Technologies, Inc. |  | TGAAAGACATTTTGATGGCATC |
| Sequence-based reagent | SALK_107965 RP | Integrated DNA Technologies, Inc. |  | CCAAATCAAGCTTTGCAAGAC |
| Sequence-based reagent | SALK_083649 pgp1-100 LP | Integrated DNA Technologies, Inc. |  | GAAGACTGCGACAAGGACAAG |
| Sequence-based reagent | SALK_083649 pgp1-100 RP | Integrated DNA Technologies, Inc. |  | GCAAGAGCGATGTTGAAGAAC |
| Sequence-based reagent | SALK_033455 atmdr1-101 LP | Integrated DNA Technologies, Inc. |  | GCAATTGCAATTCTCTGCTTC |
| Sequence-based reagent | SALK_033455 atmdr1-101 RP | Integrated DNA Technologies, Inc. |  | CTCAGGCAATTGCTCAAGTTC |
| Sequence-based reagent | SALK_012836 twd1 LP | Integrated DNA Technologies, Inc. |  | GTGAAGCTGAGGTCTTGGATG |
| Sequence-based reagent | SALK_012836 twd1 RP | Integrated DNA Technologies, Inc. |  | TATGGCCTGAAACAGCAAACC |
| Sequence-based reagent | aux1-21 Fwd | Integrated DNA Technologies, Inc. |  | CTGGAAAGCACTAGGACTCGC |
| Sequence-based reagent | aux1-21 Rev | Integrated DNA Technologies, Inc. |  | AAGCGGCGAAGAAACGATACAG |
| Sequence-based reagent | lax1 Fwd | Integrated DNA Technologies, Inc. |  | ATATGGTTGCAGGTGGCACA |
| Sequence-based reagent | lax1 WT Rev | Integrated DNA Technologies, Inc. |  | GTAACCGGCAAAAGCTGCA |
| Sequence-based reagent | lax123 mutant Rev | Integrated DNA Technologies, Inc. |  | AAGCACGACGGCTGTAGAATAG |
| Sequence-based reagent | lax2 Fwd | Integrated DNA Technologies, Inc. |  | ATGGAGAACGGTGAGAAAGCAGC |
| Sequence-based reagent | lax2 WT Rev | Integrated DNA Technologies, Inc. |  | CGCAGAAGGCAGCGTTAGCG |
| Sequence-based reagent | lax3 Fwd | Integrated DNA Technologies, Inc. |  | TACTTCACCGGAGCCACCA |
| Sequence-based reagent | lax3 WT Rev | Integrated DNA Technologies, Inc. |  | TGATTGGTCCGAAAAAGG |
| Sequence-based reagent | dSpm5 | Integrated DNA Technologies, Inc. |  | CGGGATCCGACACTCTTTAATTAACTGACACTC |
| Sequence-based reagent | SALK_020355 LP (aux1) | Integrated DNA Technologies, Inc. |  | GGCTCCCGTAAAATAAAGCAC |
| Sequence-based reagent | SALK_020355 RP (aux1) | Integrated DNA Technologies, Inc. |  | AATTATCGTTGGTTTCAGGTGG |
| Sequence-based reagent | SALK_071064 lax1 LP | Integrated DNA Technologies, Inc. |  | CAATAGTAGTCTCCGGGGAGG |
| Sequence-based reagent | SALK_071064 lax1 RP | Integrated DNA Technologies, Inc. |  | ACAACACAAGCTTGGTTGGAC |
| Sequence-based reagent | AXR1-Acc1 | Integrated DNA Technologies, Inc. |  | AAACCAACTTAACGTTTGCATGTCG |
| Sequence-based reagent | AXR1-15 | Integrated DNA Technologies, Inc. |  | TCTCATATGTACTTTTCCTCGTCCTCTTCAC |
| Sequence-based reagent | axr1-12 forw | Integrated DNA Technologies, Inc. |  | CCGAGCAGCATCCCAAAAC |
| Sequence-based reagent | axr1-12 rev | Integrated DNA Technologies, Inc. |  | GTTGGCAGCAAATCTGTCCG |
| Sequence-based reagent | AXL SAIL LP | Integrated DNA Technologies, Inc. |  | TGGACTTACTGGGTTTGTTCG |
| Sequence-based reagent | AXL SAIL RP | Integrated DNA Technologies, Inc. |  | CAAACCTTGAGTGCTGCTACC |
| Sequence-based reagent | LB3 | Integrated DNA Technologies, Inc. |  | TAGCATCTGAATTTCATAACCAATCTCGATACAC |
| Sequence-based reagent | SALK_045424 gn LP | Integrated DNA Technologies, Inc. |  | TGATCCAAATCACTGGGTTTC |
| Sequence-based reagent | SALK_045424 gn RP | Integrated DNA Technologies, Inc. |  | AGCTGAAGATAGGGAATTCGC |
| Sequence-based reagent | tir1-1F2 | Integrated DNA Technologies, Inc. |  | AGCGACGGTGATTAGGAGG |
| Sequence-based reagent | tir1-1R2 | Integrated DNA Technologies, Inc. |  | CAGGAACAACGCAGCAAAA |
| Sequence-based reagent | AFB2+F | Integrated DNA Technologies, Inc. |  | TTCTCCTTCGATCATTGTCAAC |
| Sequence-based reagent | AFB2-TR | Integrated DNA Technologies, Inc. |  | TAGCGGCAATAGAGGCAAGA |
| Sequence-based reagent | pROK-LB | Integrated DNA Technologies, Inc. |  | GGAACCACCATCAAACAGGA |
| Sequence-based reagent | GN_qFb | Integrated DNA Technologies, Inc. |  | ACTTGTCAACAGAGCTGGTAGC |
| Sequence-based reagent | GN_qRb | Integrated DNA Technologies, Inc. |  | GCTGCAAACCATCGAAAGAATC |
| Sequence-based reagent | ROC1 F | Integrated DNA Technologies, Inc. |  | CAAACCTCTTCTTCAGTCTGATAGAGA |
| Sequence-based reagent | ROC1 R | Integrated DNA Technologies, Inc. |  | GAGTGCTCATTCCTTATTTCTGGTAG |
| Sequence-based reagent | Aux_F380 | Integrated DNA Technologies, Inc. |  | CGGAAGGTGTGAAACATTCAGGTC |
| Sequence-based reagent | Aux_R380 | Integrated DNA Technologies, Inc. |  | CTTTGTGTCATGCATCCCAATCACT |
| Sequence-based reagent | Lax_F513 | Integrated DNA Technologies, Inc. |  | CGGGGGCAATGGATTTAGTATGAAG |
| Sequence-based reagent | Lax_R513 | Integrated DNA Technologies, Inc. |  | AGGCCGAGGAAAGACCAAATACG |
| Chemical compound, drug | N-1-Naphthylpthalamic acid | Chem Service, Inc. | N-12507 |  |
| Chemical compound, drug | Benzeneboronic acid | Alfa Aesar | A14257 |  |
| Chemical compound, drug | 3-Indoleacetic acid | Sigma-Aldrich | I2886 |  |
