# Supplementary material for: Coordination of tissue cell polarity by auxin transport and signaling
Source: eLife. 2019 Dec 3;8:e51061. doi: 10.7554/eLife.51061 (PMC6890459; doi:10.7554/eLife.51061)
Supplement: Supplementary file 2. — (A) Embryo viability of WT, pin3;4;7, and pin2;3;4;7. (B) Embryo viability of toz, mp, pin1, pin1,3;4;7, pin1,3;2;4;7, and pin1,3,6;4;7;8. (C) Embryo viability of pin1, pin1,3;4;7, pin1,3;2;4;7, and pin1,3,6;4;7;8. (D) Embryo viability of WT, abcb1, abcb19, abcb1;19, and twd1. (E) Embryo viability of toz, mp, pin1,3,6, and pin1,3,6;abcb1;19. (F) Embryo viability of pin1,3,6 and pin1,3,6;abcb1;19. (G) Embryo viability of WT, aux1, lax1, aux1;lax1, and aux1;lax1;2;3. (H) Embryo viability of toz, mp, pin1,3,6, and pin1,3,6;aux1;lax1. (I) Embryo viability of pin1,3,6 and pin1,3,6;aux1;lax1. (J) Embryo viability of axr1;axl, tir1;afb2, gn;pin1,3;4;7, and gn;pin1,3,6;4;7;8. (K) Embryo viability of gn;pin1,3;4;7 and gn;pin1,3,6;4;7;8. (L) Embryo viability of WT, axr1, and tir1;afb2. (M) Embryo viability of toz, mp, pin1,3,6;4;7;8, pin1,3,6;4;7;8;axr1, and pin1,3,6;4;7;8;tir1;afb2. (N) Embryo viability of pin1,3,6;4;7;8;axr1 and pin1,3,6;4;7;8;tir1;afb2. (O) Embryo viability of toz, mp, gn and gn;axr1. (P) Embryo viability of gn and gn;axr1. (Q) Genotyping strategies. (R) Light paths. [file elife-51061-supp2.pdf]

# Supplementary File 2. Supplementary Tables

## Supplementary File 2A. Embryo viability of WT, *pin3;4;7* and *pin2;3;4;7*

| **Genotype of self-fertilized parent** | **Proportion of viable embryos in siliques of self-fertilized parent (no. of non-aborted seeds / total no. of seeds)** | **Percentage of viable seeds in siliques of self-fertilized parent** |
|---|---|---|
| WT | 293/293 | 100.0 |
| *pin3/pin3;pin4/pin4;pin7/pin7* | 275/276 | 99.6 |
| *pin2/pin2;pin3/pin3;pin4/pin4;pin7/pin7* | 271/271 | 100.0 |

Difference between *pin3;4;7* and WT and between *pin2;3;4;7* and WT was not significant by Kruskal-Wallis and Mann-Whitney test with Bonferroni correction.

## Supplementary File 2B. Embryo viability of *toz*, *mp*, *pin1*, *pin1,3;4;7*, *pin1,3;2;4;7* and *pin1,3,6;4;7;8*

| Genotype of self-fertilized parent | Proportion of viable embryos in siliques of self-fertilized parent (no. of non-aborted seeds / total no. of seeds) | Percentage of viable seeds in siliques of self-fertilized parent |
|---|---|---|
| *TOZ/toz-1* | 202/278 | 72.7 |
| *MP/mp*$^{G12}$ | 264/265*** | 99.6 |
| *PIN1/pin1-1* | 254/260*** | 97.7 |
| *PIN1/pin1-134* | 257/258*** | 99.6 |
| *PIN1/pin1-1,pin3/pin3;pin4/pin4;pin7/pin7* | 269/272*** | 98.9 |
| *PIN1/pin1-134,pin3/pin3;pin4/pin4;pin7/pin7* | 280/281*** | 99.6 |
| *PIN1/pin1-1,pin3/pin3;pin2/pin2;pin4/pin4;pin7/pin7* | 276/278*** | 99.3 |
| *PIN1/pin1-1,pin3/pin3,pin6/pin6;pin4/pin4;pin7/pin7;pin8/pin8* | 266/268*** | 99.2 |

Difference between negative control for completely penetrant embryo lethality (*mp*$^{G12}$) and positive control for completely penetrant embryo lethality (*toz-1*), between *pin1-1* and *toz-1*, between *pin1-134* and *toz-1*, between *pin1-1,3;4;7* and *toz-1*, between *pin1-134,3;4;7* and *toz-1*, between *pin1-1,3;2;4;7* and *toz-1*, and between *pin1-1,3,6;4;7;8* and *toz-1* was significant at $P<0.001$ (***) by Kruskal-Wallis and Mann-Whitney test with Bonferroni correction. Difference between *pin1-1* and *mp*$^{G12}$, between *pin1-134* and *mp*$^{G12}$, between *pin1-1,3;4;7* and *mp*$^{G12}$, between *pin1-134,3;4;7* and *mp*$^{G12}$, between *pin1-1,3;2;4;7* and *mp*$^{G12}$, and between *pin1-1,3,6;4;7;8* and *mp*$^{G12}$ was not significant by Kruskal-Wallis and Mann-Whitney test with Bonferroni correction.

**Supplementary File 2C. Embryo viability of *pin1*, *pin1,3;4;7*, *pin1,3;2;4;7* and *pin1,3,6;4;7;8***

| Genotype of self-fertilized parent | Proportion of embryo-viable mutants in progeny of self-fertilized parent (no. of mutant seedlings / total no. of seedlings) | Percentage of embryo-viable mutants in progeny of self-fertilized parent |
|---|---|---|
| *PIN1/pin1-1* | 66/239 | 27.6 |
| *PIN1/pin1-134* | 53/227 | 23.3 |
| *PIN1/pin1-1,pin3/pin3;pin4/pin4;pin7/pin7* | 52/196 | 26.5 |
| *PIN1/pin1-1-134,pin3/pin3;pin4/pin4;pin7/pin7* | 56/228 | 24.6 |
| *PIN1/pin1-1,pin3/pin3;pin2/pin2;pin4/pin4;pin7/pin7* | 61/263 | 23.2 |
| *PIN1/pin1-1,pin3/pin3,pin6/pin6;pin4/pin4;pin7/pin7;pin8/pin8* | 65/260 | 25.0 |

Difference between observed and theoretical frequency distributions of embryo-viable mutants in the progeny of self-fertilized heterozygous parents was not significant by Pearson's chi-squared ($\chi^2$) goodness-of-fit test ($\alpha$=0.05, dF=1).

**Supplementary File 2D. Embryo viability of WT, *abcb1*, *abcb19*, *abcb1;19* and *twd1***

| **Genotype of self-fertilized parent** | **Proportion of viable embryos in siliques of self-fertilized parent (no. of non-aborted seeds / total no. of seeds)** | **Percentage of viable seeds in siliques of self-fertilized parent** |
|---|---|---|
| WT | 294/294 | 100 |
| *abcb1/abcb1* | 269/272 | 98.9 |
| *abcb19/abcb19* | 271/276 | 98.2 |
| *abcb1/abcb1;abcb19/abcb19* | 276/332*** | 83.1 |
| *twd1/twd1* | 245/265*** | 92.4 |

Difference between *abcb1;19* and WT, and between *twd1* and WT was significant at $P<0.001$ (***). and between *abcb1* and WT, and between *abcb19* and WT was not significant by Kruskal-Wallis and Mann-Whitney test with Bonferroni correction.

## Supplementary File 2E. Embryo viability of *toz*, *mp*, *pin1,3,6* and *pin1,3,6;abcb1;19*

| Genotype of self-fertilized parent | Proportion of viable embryos in siliques of self-fertilized parent (no. of non-aborted seeds / total no. of seeds) | Percentage of viable seeds in siliques of self-fertilized parent |
|---|---|---|
| *TOZ/toz-1* | 202/277 | 72.9 |
| *MP/mp*$^{G12}$ | 255/256$^{***}$ | 99.6 |
| *PIN1/pin1-1,pin3/pin3,PIN6/pin6* | 263/266$^{***}$ | 98.9 |
| *PIN1/pin1-1,PIN3/pin3,PIN6/pin6;abcb1/abcb1;abcb19/abcb19* | 240/284$^{*/***}$ | 84.5 |

Difference between negative control for completely penetrant embryo lethality (*mp*$^{G12}$) and positive control for completely penetrant embryo lethality (*toz-1*) and between *pin1-1,3,6* and *toz-1* was significant at $P<0.001$ (***), and between *pin1-1,3,6;abcb1;19* and *toz-1* was significant at $P<0.05$ (*) by Kruskal-Wallis and Mann-Whitney test with Bonferroni correction. Difference between *pin1-1,3,6;abcb1;19* and *mp*$^{G12}$ was significant at $P<0.001$ (***), and between *pin1-1,3,6* and *mp*$^{G12}$ was not significant by Kruskal-Wallis and Mann-Whitney test with Bonferroni correction. Linkage in *cis* between *pin1-1* and *pin6* in *PIN1/pin1-1,pin3/pin3,PIN6/pin6* was confirmed by phenotyping the progeny of the self-fertilized *PIN1/pin1-1,pin3/pin3,PIN6/pin6* plants used for the embryo viability analysis for the presence of seedlings with cup-shaped cotyledons, which are characteristic of *pin1,6* double homozygous mutant (Sawchuk et al., 2013). Linkage in *cis* between *pin1-1*, *pin3* and *pin6* in *PIN1/pin1-1,PIN3/pin3,PIN6/pin6;abcb1/abcb1;abcb19/abcb19* was confirmed by phenotyping the progeny of the self-fertilized *PIN1/pin1-1,PIN3/pin3,PIN6/pin6;abcb1/abcb1;abcb19/abcb19* plants used for the embryo viability analysis for the presence of seedlings with cup-shaped cotyledons and by genotyping those cup-shaped-cotyledon seedling for the *pin3* mutation.

**Sawchuk, M. G., Edgar, A. and Scarpella, E.** (2013). Patterning of leaf vein networks by convergent auxin transport pathways. *PLoS Genet.* **9**, e1003294.

## Supplementary File 2F. Embryo viability of ***pin1,3,6*** and ***pin1,3,6;abcb1;19***

| **Genotype of self-fertilized parent** | **Proportion of embryo-viable mutants in progeny of self-fertilized parent (no. of mutant seedlings / total no. of seedlings)** | **Percentage of embryo-viable mutants in progeny of self-fertilized parent** |
|---|---|---|
| *PIN1/pin1-1,pin3/pin3,PIN6/pin6* | 80/361 | 22.2 |
| *PIN1/pin1-1,PIN3/pin3,PIN6/pin6;abcb1/abcb1;abcb19/abcb19* | 74/335 | 22.1 |

Difference between observed and theoretical frequency distributions of embryo-viable mutants in the progeny of self-fertilized heterozygous parents was not significant by Pearson's chi-squared (χ2) goodness-of-fit test (α=0.05, dF=1). Genotype of the mutants seedlings of *PIN1/pin1-1,pin3/pin3,PIN6/pin6* was confirmed by genotyping all mutant seedlings for *pin1-1* and *pin6* mutation. Genotype of the mutants seedlings of *PIN1/pin1-1,PIN3/pin3,PIN6/pin6;abcb1/abcb1;abcb19/abcb19* was confirmed by genotyping all mutant seedlings for *pin1-1*, *pin3* and *pin6* mutation.

**Supplementary File 2I. Embryo viability of *pin1,3,6* and *pin1,3,6;aux1;lax1***

| Genotype of self-fertilized parent | Proportion of embryo-viable mutants in progeny of self-fertilized parent (no. of mutant seedlings / total no. of seedlings) | Percentage of embryo-viable mutants in progeny of self-fertilized parent |
|---|---|---|
| *PIN1/pin1-1,pin3/pin3,PIN6/pin6* | 87/390 | 22.3 |
| *PIN1/pin1-1,pin3/pin3,PIN6/pin6;aux1/aux1-355;lax1/lax1-064* | 109/489 | 22.3 |

Difference between observed and theoretical frequency distributions of embryo-viable mutants in the progeny of self-fertilized heterozygous parents was not significant by Pearson's chi-squared ($\chi 2$) goodness-of-fit test ($\alpha$=0.05, dF=1). Genotype of the mutants seedlings of both *PIN1/pin1-1,pin3/pin3,PIN6/pin6* and *PIN1/pin1-1,pin3/pin3,PIN6/pin6;aux1/aux1-355;lax1/lax1-064* was confirmed by genotyping all mutant seedlings for *pin1-1* and *pin6* mutation.

## Supplementary File 2H. Embryo viability of ***toz***, ***mp***, ***pin1,3,6*** and ***pin1,3,6;aux1;lax1***

| **Genotype of self-fertilized parent** | **Proportion of viable embryos in siliques of self-fertilized parent (no. of non-aborted seeds / total no. of seeds)** | **Percentage of viable seeds in siliques of self-fertilized parent** |
|---|---|---|
| *TOZ/toz-1* | 185/244*** | 75.8 |
| *MP/mp$^{G12}$* | 220/220 | 100 |
| *PIN1/pin1-1,pin3/pin3,PIN6/pin6* | 259/261*** | 99.2 |
| *PIN1/pin1-1,pin3/pin3,PIN6/pin6;aux1/aux1-355;lax1/lax1-064* | 280/282*** | 99.3 |

Difference between negative control for completely penetrant embryo lethality (*mp$^{G12}$*) and positive control for completely penetrant embryo lethality (*toz-1*), between *pin1-1,3,6* and *toz-1*, and between *pin1-1,3,6;aux1-355;lax1-064* and *toz-1* was significant at $P<0.001$ (***) by Kruskal-Wallis and Mann-Whitney test with Bonferroni correction. Difference between *pin1-1,3,6* and *mp$^{G12}$*, and between *pin1-1,3,6;aux1-355;lax1-064* and *mp$^{G12}$* was not significant by Kruskal-Wallis and Mann-Whitney test with Bonferroni correction. Linkage in *cis* between *pin1-1* and *pin6* in *PIN1/pin1-1,pin3/pin3,PIN6/pin6* and *PIN1/pin1-1,pin3/pin3,PIN6/pin6;aux1/aux1-355;lax1/lax1-064* was confirmed by phenotyping the progeny of the self-fertilized plants used for the embryo viability analysis for the presence of seedlings with cup-shaped cotyledons, which are characteristic of *pin1,6* double homozygous mutant (Sawchuk et al., 2013).

**Sawchuk, M. G., Edgar, A. and Scarpella, E.** (2013). Patterning of leaf vein networks by convergent auxin transport pathways. *PLoS Genet.* **9**, e1003294.

## Supplementary File 2I. Embryo viability of *pin1,3,6* and *pin1,3,6;aux1;lax1*

| Genotype of self-fertilized parent | Proportion of embryo-viable mutants in progeny of self-fertilized parent (no. of mutant seedlings / total no. of seedlings) | Percentage of embryo-viable mutants in progeny of self-fertilized parent |
|---|---|---|
| *PIN1/pin1-1,pin3/pin3,PIN6/pin6* | 87/390 | 22.3 |
| *PIN1/pin1-1,pin3/pin3,PIN6/pin6;aux1/aux1-355;lax1/lax1-064* | 109/489 | 22.3 |

Difference between observed and theoretical frequency distributions of embryo-viable mutants in the progeny of self-fertilized heterozygous parents was not significant by Pearson's chi-squared ($\chi 2$) goodness-of-fit test ($\alpha$=0.05, dF=1). Genotype of the mutants seedlings of both *PIN1/pin1-1,pin3/pin3,PIN6/pin6* and *PIN1/pin1-1,pin3/pin3,PIN6/pin6;aux1/aux1-355;lax1/lax1-064* was confirmed by genotyping all mutant seedlings for *pin1-1* and *pin6* mutation.

**Supplementary File 2G. Embryo viability of WT, *aux1*, *lax1*, *aux1*;*lax1* and *aux1*;*lax1*;*2*;*3***

| Genotype of self-fertilized parent | Proportion of viable embryos in siliques of self-fertilized parent (no. of non-aborted seeds / total no. of seeds) | Percentage of viable seeds in siliques of self-fertilized parent |
|---|---|---|
| WT | 272/274 | 99.3 |
| *aux1/aux1-355* | 266/267 | 99.6 |
| *lax1/lax1-064* | 265/267 | 99.2 |
| *aux1/aux1-355*;*lax1/lax1-064* | 278/281 | 98.9 |
| *aux1/aux1-21*;*lax1/lax1*;*lax2/lax2-1*;*lax3/lax3* | 261/262 | 99.6 |

Difference between *aux1-355* and WT, between *lax1-064* and WT, between *aux1-355*;*lax1-064* and WT, and between *aux1-21*;*lax1*;*2-1*;*3* and WT was not significant by Kruskal-Wallis and Mann-Whitney test with Bonferroni correction.

**Supplementary File 2F. Embryo viability of *pin1,3,6* and *pin1,3,6;abcb1;19***

| **Genotype of self-fertilized parent** | **Proportion of embryo-viable mutants in progeny of self-fertilized parent (no. of mutant seedlings / total no. of seedlings)** | **Percentage of embryo-viable mutants in progeny of self-fertilized parent** |
|---|---|---|
| *PIN1/pin1-1,pin3/pin3,PIN6/pin6* | 80/361 | 22.2 |
| *PIN1/pin1-1,PIN3/pin3,PIN6/pin6;abcb1/abcb1;abcb19/abcb19* | 74/335 | 22.1 |

Difference between observed and theoretical frequency distributions of embryo-viable mutants in the progeny of self-fertilized heterozygous parents was not significant by Pearson's chi-squared ($\chi 2$) goodness-of-fit test ($\alpha$=0.05, dF=1). Genotype of the mutants seedlings of *PIN1/pin1-1,pin3/pin3,PIN6/pin6* was confirmed by genotyping all mutant seedlings for *pin1-1* and *pin6* mutation. Genotype of the mutants seedlings of *PIN1/pin1-1,PIN3/pin3,PIN6/pin6;abcb1/abcb1;abcb19/abcb19* was confirmed by genotyping all mutant seedlings for *pin1-1*, *pin3* and *pin6* mutation.

**Supplementary File 2I. Embryo viability of *pin1,3,6* and *pin1,3,6;aux1;lax1***

| Genotype of self-fertilized parent | Proportion of embryo-viable mutants in progeny of self-fertilized parent (no. of mutant seedlings / total no. of seedlings) | Percentage of embryo-viable mutants in progeny of self-fertilized parent |
|---|---|---|
| *PIN1/pin1-1,pin3/pin3,PIN6/pin6* | 87/390 | 22.3 |
| *PIN1/pin1-1,pin3/pin3,PIN6/pin6;aux1/aux1-355;lax1/lax1-064* | 109/489 | 22.3 |

Difference between observed and theoretical frequency distributions of embryo-viable mutants in the progeny of self-fertilized heterozygous parents was not significant by Pearson's chi-squared (χ2) goodness-of-fit test (α=0.05, dF=1). Genotype of the mutants seedlings of both *PIN1/pin1-1,pin3/pin3,PIN6/pin6* and *PIN1/pin1-1,pin3/pin3,PIN6/pin6;aux1/aux1-355;lax1/lax1-064* was confirmed by genotyping all mutant seedlings for *pin1-1* and *pin6* mutation.

**Supplementary File 2J. Embryo viability of *axr1*;*axl*, *tir1*;*afb2*, *gn*;*pin1*,*3*;*4*;*7* and *gn*;*pin1*,*3*,*6*;*4*;*7*;*8***

| Genotype of self-fertilized parent | Proportion of viable embryos in siliques of self-fertilized parent (no. of non-aborted seeds / total no. of seeds) | Percentage of viable seeds in siliques of self-fertilized parent |
|---|---|---|
| *AXR1/axr1-12*;*AXL/axl* | 900/978 | 92 |
| *TIR1/tir1*;*AFB2/afb2* | 777/781*** | 99.5 |
| *GN/gn-13*;*PIN1/pin1-1*,*pin3/pin3*;*pin4/pin4*;*pin7/pin7* | 482/484*** | 99.6 |
| *GN/gn-13*;*PIN1/pin1-1*,*pin3/pin3*,*pin6/pin6*;*pin4/pin4*;*pin7/pin7*;*pin8/pin8* | 571/575*** | 99.3 |

Difference between negative control for completely penetrant embryo lethality (*tir1*;*afb2*) and positive control for completely penetrant embryo lethality (*axr1-12*;*axl*), between *gn*;*pin1-1*,*3*;*4*;*7* and *axr1-12*;*axl*, and between *gn*;*pin1-1*,*3*,*6*;*4*;*7*;*8* and *axr1-12*;*axl* was significant at $P<0.001$ (***) by Kruskal-Wallis and Mann-Whitney test with Bonferroni correction. Difference between *gn*;*pin1-1*,*3*;*4*;*7* and *tir1*;*afb2*, and between *gn*;*pin1-1*,*3*,*6*;*4*;*7*;*8* and *tir1*;*afb2* was not significant by Kruskal-Wallis and Mann-Whitney test with Bonferroni correction.

## Supplementary File 2K. Embryo viability of ***gn*;*pin1*,*3*;*4*;*7*** and ***gn*;*pin1*,*3*,*6*;*4*;*7*;*8***

| **Genotype of self-fertilized parent** | **Proportion of embryo-viable mutants in progeny of self-fertilized parent (no. of mutant seedlings / total no. of seedlings)** | **Percentage of embryo-viable mutants in progeny of self-fertilized parent** |
|---|---|---|
| *GN/gn-13*;*PIN1/pin1-1*,*pin3/pin3*;*pin4/pin4*;*pin7/pin7* | 256/3624 | 7.1 |
| *GN/gn-13*;*PIN1/pin1-1*,*pin3/pin3*,*pin6/pin6*;*pin4/pin4*;*pin7/pin7*;*pin8/pin8* | 222/3231 | 6.9 |

Difference between observed and theoretical frequency distributions of embryo-viable mutants in the progeny of self-fertilized heterozygous parents was not significant by Pearson's chi-squared ($\chi^2$) goodness-of-fit test ($\alpha$=0.05, dF=1).

**Supplementary File 2L. Embryo viability of WT, *axr1* and *tir1*;*afb2***

| **Genotype of self-fertilized parent** | **Proportion of viable embryos in siliques of self-fertilized parent (no. of non-aborted seeds / total no. of seeds)** | **Percentage of viable seeds in siliques of self-fertilized parent** |
|---|---|---|
| WT | 408/412 | 99 |
| *axr1-3* | 391/403 | 97 |
| *tir1*;*afb2* | 300/303 | 99 |

Difference between *axr1-3* and WT, and between *tir1*;*afb2* and WT was not significant by Kruskal-Wallis and Mann-Whitney test with Bonferroni correction.

**Supplementary File 2M. Embryo viability of *toz*, *mp*, *pin1,3,6;4;7;8*, *pin1,3,6;4;7;8;axr1*, *pin1,3,6;4;7;8;tir1;afb2***

| **Genotype of self-fertilized parent** | **Proportion of viable embryos in siliques of self-fertilized parent (no. of non-aborted seeds / total no. of seeds)** | **Percentage of viable seeds in siliques of self-fertilized parent** |
|---|---|---|
| *TOZ/toz-1* | 190/239 | 79.5 |
| *MP/mp*$^{G12}$ | 261/262$^{***}$ | 99.6 |
| *PIN1/pin1-1,pin3/pin3,pin6/pin6;pin4/pin4;pin7/pin7;pin8/pin8* | 243/244$^{***}$ | 99.6 |
| *PIN1/pin1-1,pin3/pin3,pin6/pin6;pin4/pin4;pin7/pin7;pin8/pin8;axr1/axr1-3* | 240/248$^{***}$ | 96.8 |
| *PIN1/pin1-1,pin3/pin3,pin6/pin6;pin4/pin4;pin7/pin7;pin8/pin8;tir1/tir1;afb2/afb2* | 473/475$^{***}$ | 99.6 |

Difference between negative control for completely penetrant embryo lethality (*mp*$^{G12}$) and positive control for completely penetrant embryo lethality (*toz-1*), between *pin1-1,3,6;4;7;8* and *toz-1*, between *pin1-1,3,6;4;7;8;axr1-3* and *toz-1*, and between *pin1-1,3,6;4;7;8;tir1;afb2* and *toz-1* was significant at $P<0.001$ (***) by Kruskal-Wallis and Mann-Whitney test with Bonferroni correction. Difference between *pin1-1,3,6;4;7;8* and *mp*$^{G12}$, between *pin1-1,3,6;4;7;8;axr1-3* and *mp*$^{G12}$, and between *pin1-1,3,6;4;7;8;tir1;afb2* and *mp*$^{G12}$ was not significant by Kruskal-Wallis and Mann-Whitney test with Bonferroni correction.

**Supplementary File 2N. Embryo viability of *pin1,3,6;4;7;8;axr1* and *pin1,3,6;4;7;8;tir1;afb2***

| Genotype of self-fertilized parent | Proportion of embryo-viable mutants in progeny of self-fertilized parent (no. of mutant seedlings / total no. of seedlings) | Percentage of embryo-viable mutants in progeny of self-fertilized parent |
|---|---|---|
| *PIN1/pin1-1,pin3/pin3,pin6/pin6;pin4/pin4;pin7/pin7;pin8/pin8;axr1/axr1-3* | 66/277 | 23.8 |
| *PIN1/pin1-1,pin3/pin3,pin6/pin6;pin4/pin4;pin7/pin7;pin8/pin8;tir1/tir1;afb2/afb2* | 77/324 | 23.8 |

Difference between observed and theoretical frequency distributions of embryo-viable mutants in the progeny of self-fertilized heterozygous parents was not significant by Pearson's chi-squared ($\chi^2$) goodness-of-fit test ($\alpha$=0.05, dF=1).

**Supplementary File 2O. Embryo viability of *toz*, *mp*, *gn* and *gn*;*axr1***

| Genotype of self-fertilized parent | Proportion of viable embryos in siliques of self-fertilized parent (no. of non-aborted seeds / total no. of seeds) | Percentage of viable seeds in siliques of self-fertilized parent |
|---|---|---|
| *TOZ/toz-1* | 206/259 | 79.5 |
| *MP/mp*$^{G12}$ | 243/247*** | 98.4 |
| *GN/gn-13* | 248/252*** | 98.4 |
| *GN/gn-13*;*axr1/axr1-3* | 264/270*** | 97.8 |
| *GN/gn-13*;*axr1/axr1-12* | 214/224*** | 95.6 |

Difference between negative control for completely penetrant embryo lethality (*mp*$^{G12}$) and positive control for completely penetrant embryo lethality (*toz-1*), between *gn-13* and *toz-1*, between *gn-13*;*axr1-3* and *toz-1*, and between *gn-13*;*axr1-12* and *toz-1* was significant at $P<0.001$ (***) by Kruskal-Wallis and Mann-Whitney test with Bonferroni correction. Difference between *gn-13* and *mp*$^{G12}$, between *gn-13*;*axr1-3* and *mp*$^{G12}$, and between *gn-13*;*axr1-12* and *mp*$^{G12}$ was not significant by Kruskal-Wallis and Mann-Whitney test with Bonferroni correction.

## Supplementary File 2P. Embryo viability of *gn* and *gn*;*axr1*

| Genotype of self-fertilized parent | Proportion of embryo-viable mutants in progeny of self-fertilized parent (no. of mutant seedlings / total no. of seedlings) | Percentage of embryo-viable mutants in progeny of self-fertilized parent |
|---|---|---|
| *GN/gn-13* | 101/411 | 24.6 |
| *GN/gn1-13*;*axr1-3* | 74/321 | 23.0 |
| *GN/gn1-13*;*axr1-12* | 70/276 | 25.4 |

Difference between observed and theoretical frequency distributions of embryo-viable mutants in the progeny of self-fertilized heterozygous parents was not significant by Pearson's chi-squared ($\chi^2$) goodness-of-fit test ($\alpha$=0.05, dF=1).

## Supplementary File 2Q. Genotyping strategies

| Line | Strategy |
|---|---|
| *gn-13* | *GN*: 'SALK_045424 gn LP' and 'SALK_045424 gn RP'; *gn*: 'SALK_045424 gn RP' and 'LBb1.3' |
| *gn-18* | *GN*: 'Salk026031 LP gnp close' and 'Salk026031 RP gnp close'; *gn*: 'Salk026031 RP gnp close' and 'LBb1.3' |
| *fwr* ($gn^{fwr}$) | 'FWR for' and 'FWR REV2'; *EcoR*I |
| *van7/emb30-7* ($gn^{van7}$) | 'van7 Hpa1 FP' and 'van7 Hpa1 RP'; *Hpa*I |
| *pin1-1* | 'pin1-1 F' and 'pin1-1 R'; *Tat*I |
| *pin1-134* | 'pin1-1 F' and 'pin1-134 R mse-I'; *Mse*I |
| *pin3-3* | 'pin3-3 F' and 'pin3-3 R'; *Sty*I |
| *pin4-2* | *PIN4*: 'PIN4 forw geno II' and 'PIN4en rev Ikram'; *pin4*: 'PIN4en rev Ikram' and 'en primer' |
| $pin7^{En}$ | *PIN7*: 'PIN7en forw Ikram' and 'PIN7en rev'; *pin7*: 'PIN7en rev Ikram II' and 'en primer' |
| *eir1-1* (*pin2*) | 'eir1-1 F' and 'eir1-1 R'; *Bse*LI |
| *pin6* | *PIN6*: 'PIN6 spm F' and 'PIN6 spm R'; *pin6*: 'PIN6 spm F' and 'Spm32' |
| *pin8-1* | *PIN8*: 'SALK_107965 LP' and 'SALK_107965 RP'; *pin8*: 'SALK_107965 RP' and 'LBb1.3' |
| *pgp1-100* (*abcb1*) | *ABCB1*: 'SALK_083649 pgp1-100 LP' and 'SALK_083649 pgp1-100 RP'; *abcb1*: 'SALK_083649 pgp1-100 RP' and 'LBb1.3' |
| *mdr1-101* (*abcb19*) | *ABCB19*: 'SALK_033455 atmdr1-101 LP' and 'SALK_033455 atmdr1-101 RP'; *abcb19*: 'SALK_033455 atmdr1-101 RP' and 'LBb1.3' |
| *ucu2-4* (*twd1*) | *UCU2*: 'SALK_012836 twd1 LP' and 'SALK_012836 twd1 RP'; *ucu2*: 'SALK_012836 twd1 RP' and 'LBb1.3' |
| *aux1-21* | 'aux1-21 Fwd' and 'aux1-21 Rev'; *ApaL*I |
| *lax1* | *LAX1*: 'lax1 Fwd' and 'lax1 WT Rev'; *lax1*: 'lax1 fwd' and 'lax123 mutant Rev' |
| *lax2-1* | *LAX2*: 'lax2 Fwd' and 'lax2 WT Rev'; *lax2*: 'lax2 fwd' and 'lax123 mutant Rev' |
| *lax3* | *LAX3*: 'lax3 Fwd' and 'lax3 WT Rev'; *lax3*: 'lax3 fwd' and 'dSpm5' |
| *aux1-355* | *AUX1*: 'SALK_020355 LP (aux1)' and 'SALK_020355 RP (aux1)'; *aux1*: 'SALK_020355 RP (aux1)' and 'LBb1.3' |
| *lax1-064* | *LAX1*: 'SALK_071064 lax1 LP' and 'SALK_071064 lax1 RP'; *lax1*: 'SALK_071064 lax1 RP' and 'LBb1.3' |
| *axr1-3* | 'AXR1-Acc1' and 'AXR1-15'; *Sal*I |
| *axr1-12* | 'axr1-12 forw' and 'axr1-12 rev'; *Dra*I |
| *axl* | *AXL*: 'AXL SAIL LP' and 'AXL SAIL RP'; *axl*: 'AXL SAIL RP' and 'LB3' |
| *tir1-1* | 'tir1-1F2' and 'tir1-1R2', *Bsa*I |
| *afb2-3* | *AFB2*: 'AFB2+F' and 'AFB2-TR'; *afb2*: 'pROK-LB' and 'AFB2-TR' |

## Supplementary File 2R. Light paths

| Fluorophore | Laser | Wavelength (nm) | Main dichroic beam splitter | First secondary dichroic beam splitter | Second secondary dichroic beam splitter | Emission filter (detector) |
|---|---|---|---|---|---|---|
| YFP | Ar | 514 | HFT 405/514/594 | NFT 595 | NFT 515 | BP 520–555 IR (PMT3) |
| GFP; Autofluorescence | Ar | 488 | HFT 405/488/594 | NFT 545 | NFT 490 (PMT3); Plate (META) | BP 505–530 (PMT3); 550–574 (META) |
| GFP | Ar | 488 | HFT 405/488/594 | NFT 545 | NFT 490 | BP 505–530 (PMT3) |
| Lignin | Diode | 405 | HFT 405/514/594 | Mirror | NFT 490 | BP 420–480 (PMT2) |
